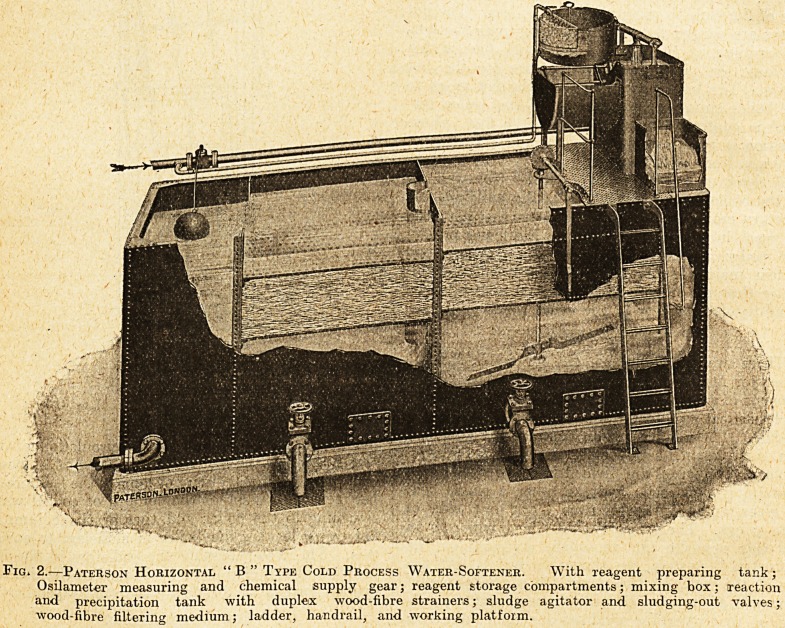# Ham Green Hospital and Sanatorium, Bristol

**Published:** 1916-11-04

**Authors:** 


### THE HEATING OF HOSPITALS.

### Ham Green Hospital and Sanatorium, Bristol.

The Ham Green Hospital belongs to the Bristol Corporation, and is situated some miles beyond the city boundary, and at a distance from the nearest village. It consists of six hospital blocks, each of two storeys, an administration block, and the recently erected single-storey sanatorium. Four of the hospital blocks and the administration block are warmed by coal fires in the ordinary type of fireplace; the other two hospital blocks and the sanatorium are warmed by hot-water radiators and hot-water pipes. Each hospital block and each of the two sanatorium buildings has its own hot-water boiler. In the hospital blocks the hot-water boilers are of the Goliath pattern tubular boilers. The hot gases are caused to make two turns over the hot-water pipes by the aid of a brickwork flue, which encloses the pipes, but allows space for the hot gases. The brickwork flue leads the hot gases to the boiler chimney; the draught of the boiler furnace is controlled by a damper in the flue, whose position is regulated by a weight and a system of levers in front of the boiler.

A rising main carries the hot water from the top of the boiler to the upper floor of the building, and a return main brings the cooled water that has done its work in the radiators back to the lower part of the boiler. Branch pipes between the flow and return mains supply the radiators. The flow of water through individual radiators is controlled by hand-regulated valves in the usual way.

### The Sanatorium.

There are two buildings, one for males and the other for females, constructed exactly alike. Each consists of a long, rather low building, with what an ecclesiastical architect would call a transept in the middle. The middle portion contains the kitchens, the duty room, bath-rooms, and some patients' rooms. The wings are divided into two wards, one containing eight beds and the other a single bed. A glass-roofed verandah runs along one side of both wings. The patients' rooms have windows on the side opposite the verandah, arranged on the "Austral" system, as shown in fig. 1. At the top of the wall, on the other side of the patients' rooms, is an opening between the wall and the ceiling, so that when the windows are open there is a current of air passing right across the room. The patients' rooms are heated by two 3-in. pipes running right along the floor against the wall on the side opposite to the verandah under the windows. The single bedrooms are heated by single radiators placed under the windows; the duty room is heated partly by an open fire, in the usual modern grate, and partly by a radiator on the opposite side of the room. The "acute" block, arranged for eight beds, is warmed by four radiators placed against three of the walls. The radiators and the 3-in. pipes are heated by hot water supplied by sectional Robin Hood boilers fixed in the basement of each building. The sectional hot-water boiler is one that is used very much in hot-water heating. It is made to any size in sections, each section consisting of what are practically square tubes, forming three sides of a rectangle. The water to be heated is contained in the tubes, and the grate for the fuel forms the fourth side of the rectangle. The front of the boiler is closed by the furnace door and the back by a casting carrying the first

Fig. 1.—Austral Window in Ham Green Sanatorium.

length of the chimney. The circulation of the water through the radiators and the 3-in. pipes is on the thermo-syphon system; the difference between the weight of the column of cooled water returning to the boiler and that of the column of hot water leaving the boiler is sufficient to ensure this. The water heating the radiators in the two hospital blocks also flows by the aid of the thermo-syphon formed by the flow and return pipes.

### The Steam Plant.

In addition to the hot-water boilers supplying the heating systems, there are two Cornish boilers supplying steam for the purposes named below. Each is 22 feet long and 5 feet 6 inches in diameter, and is arranged to work up to 80 lb. per square inch pressure. One boiler is sufficient to provide all the steam required; the other one is kept as spare. The Cornish boiler differs from the Lancashire boiler that is so much used in the North in having only one furnace and one main flue in place of two. In both there is a long containing cylinder, which is partly filled with water and partly with steam when the boiler is at work; the Lancashire boiler has two smaller cylinders, running from end to end of the boiler; furnaces are fixed at one end of each tube, and the hot gases formed by the burning coal flow through the tubes, and then round outside of the boiler, in flues provided by the brickwork setting in which the boiler rests. In the Cornish boiler there is one such cylinder, larger than either of the two cylinders in the Lancashire boiler, running from end to end of the containing cylinder, and having a furnace at one end.

A small portion of the steam furnished by the boiler is employed in a low-pressure steam radiator, fixed in the resident medical officer's laboratory; the pressure of the steam is reduced to 10 lb. per square inch by a valve, and the water formed by condensation in the radiator is carried off by a steam trap.

9 p.m., the accumulator then takes charge; the accumulator receives its own charge from the dynamo during the day. The exhaust steam from the engine is used to heat the feed-water for the boiler in a Boyle's feed-water heater. Steam is used in the washing machines and also in the drying closet; the pressure is reduced to 30 lb. per square inch before entering the steam coil. The clothes in the drying closets are subject to a current of air warmed by passing over the steam coils referred to above, the circulation of the air being accomplished by a 2-feet Blackman fan, driven from the same shaft as the laundry machinery.

### Water for the Hospital.

All the water in the neighbourhood of Bristol

Fig. 2.—Paterson Horizontal "B" Type Cold Process Water-Softener. With reagent preparing tank; Osilameter measuring and chemical supply gear; reagent storage compartments; mixing box; reaction and precipitation tank with duplex wood-fibre strainers; sludge agitator and sludging-out valves; wood-fibre filtering medium; ladder, handrail, and working platform.

The major portion of the steam from the boiler is used in a Bumsted-Chandler engine of about 50 horse-power, which drives the electric-light generator and also the laundry machinery. The shaft of the engine is directly coupled to the shaft of a Crompton compound-wound continuous-current dynamo, running at 400 revolutions per minute, and furnishing 200 amperes at 130 volts. There are two engines and two dynamos; one is sufficient to do all the work, the other is spare.

The whole of the buildings are lighted by metallic-filament incandescent lamps. For furnishing current during the night a battery of accumulators is provided. In winter the dynamo runs until and for a good many miles round is very hard, and consequently a water-softening apparatus is required where large quantities are used. At Ham Green a Paterson water-softener is used, of the type shown in fig. 2; measured quantities of lime and carbonate of soda are mixed with measured quantities of water, the treated water passing down into the precipitation tanks, shown at the bottom of the figure, where the separation of the substances to which hardness is due is completed. The purified water passes upwards through the wood-fibre filters shown, and is pumped to an overhead tank, from which it flows by gravity, and, assisted by the boiler feed-pump, finds its way into the boiler.

## Figures and Tables

**Figure f1:**
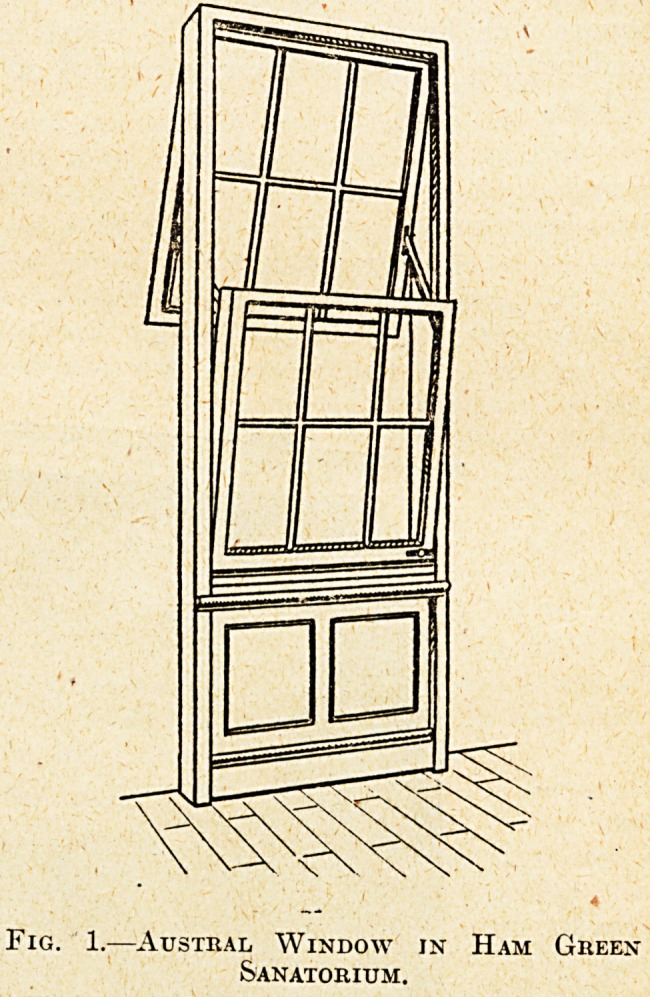


**Figure f2:**